# Primary Stability of Three Different Osteotomy Techniques in Medullary Bone: An in Vitro Study

**DOI:** 10.3390/dj8010021

**Published:** 2020-02-21

**Authors:** Ferdinando Attanasio, Alessandro Antonelli, Ylenia Brancaccio, Fiorella Averta, Michele Mario Figliuzzi, Leonzio Fortunato, Amerigo Giudice

**Affiliations:** Department of Health Sciences, School of Dentistry, Magna Græcia University of Catanzaro, 88100 Catanzaro, Italy; ferdinando.attanasio@gmail.com (F.A.); ylenia.brancaccio@studenti.unicz.it (Y.B.); fiorella.averta@studenti.unicz.it (F.A.); figliuzzi@unicz.it (M.M.F.); leo@unicz.it (L.F.); a.giudice@unicz.it (A.G.)

**Keywords:** primary implant stability, in vitro study, osteotomy, resonance frequency analysis, insertion torque

## Abstract

Background: The aim of this in vitro study was to analyse the primary stability of 20 implants placed with Twist drills (TD) versus 20 implants placed with Summers osteotomes (SO) and 20 implants placed with B&B bone compactors (BC) in medullary bone (quality type III and type IV). Methods: The implants were placed in 10 fresh pig ribs fixed on a bench vice in order to avoid micro-movements during surgical procedures and measure recording. Peak insertion torque (PIT) and Peak removal torque (PRT) were recorded with MGT-12 digital torque gauge and ISQ was recorded through OSSTELL ISQ™ device by an independent operator. Results: Comparing our data (Tukey test *p* = 0.05), it was evidenced a statistically significant difference in the PIT between TD and BC groups (*p* = 0.01). Analysing ISQ data, there was a statistically significant difference between the TD and BC groups (*p* = 0.0001) and between the SO and BC groups (*p* = 0.014). The analysis of PRT evidenced a statistically significant difference between the TD and BC groups (*p* = 0.038). Conclusions: This study evidenced that bone compactor preparation can positively influence primary implant stability (PS), however further in vivo studies and a larger sample are necessary to assess the usefulness in several clinical settings.

## 1. Introduction

Endosteal implants are used in many medical procedures, from dental implants to orthopaedic surgery. These devices allow the rehabilitation of damaged tissue caused by trauma and pathology [1]. The main goal of an implant rehabilitation is to achieve osseo-integration. One of the most important features allowing for osseo-integration is primary implant stability (PS). PS can be defined as the mechanical stability between the implant and the bone surrounding the fixture [2]. Primary implant stability depends on the quantity and quality of the bone, the fixture design, and the surgical technique used [3]. Previous literature suggests that PS is achieved when the micro-motion of the implant has less than 50- to 150-μm thresholds before osseo-integration occurs [4,5]. There are many parameters that evaluate the PS; among these insertion and removal torque are parameters that are mainly used to analyse the degree of PS both on animals both on synthetic bone [6]. In the last few decades, a wide variety of shapes, platforms, and designs have been tried, tested, and marketed [7,8], in order to achieve or improve primary stability. The most common osteotomy surgical technique for implant placement is the bone drilling protocol [7]. However, several protocols were developed to perform implant site preparation without bone drilling, particularly for in poor density bone. The Summers’ osteotomy technique was introduced [9] to increase primary stability and expand the edentulous ridge without the drilling of bone tissue. This technique allows for the creation of a layer of compacted bone at the bone-implant interface in the medullary bone [10]. This surgical practice can improve the primary stability of the implant. However, drawbacks in this technique include surgical trauma, unintentional fracture or bone displacement, and even vertigo in the patient [11]. Recently, a new osteotomy technique proposed by B&B dental implant (San Benedetto, BO, Italy) has been introduced. Through the use of customized compactors, this surgical technique causes the lateral thrust of the bone surrounding the implant fixture without destroy the bone matrix component, while taking advantage of the elasticity of the bone to deform slowly. The aim of this study is to investigate the usefulness of these new compactors in increasing the degree of primary implant stability in medullary bone. Objectives: To compare the Implant Stability Quotient (ISQ) and insertion torque value of 20 implants placed with Twist drills (TD) against 20 implant sites prepared with Summers osteotomes (SO) and 20 implant sites prepared with B&B bone compactors (BC) placed in in medullary bone (type III and IV).

## 2. Materials and Methods

This in vitro study was carried out in the Dental clinic of The Magna Graecia University.

**Figure 1:**
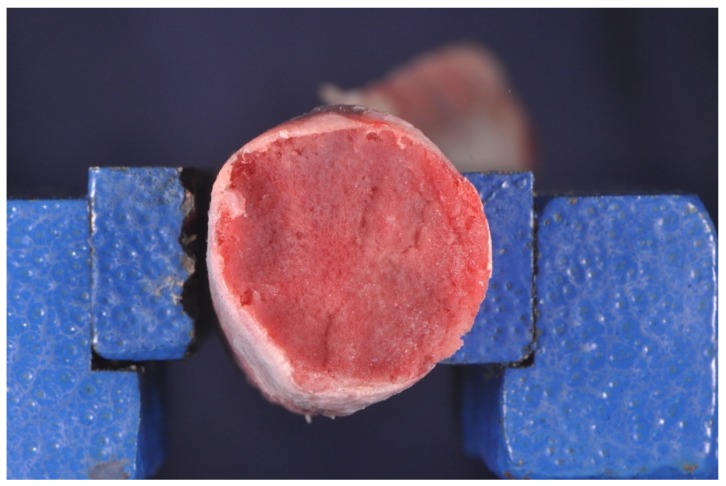
Fresh pig rib sample. The most distal region of the rib is smaller in diameter and has a great percentage of bone marrow.

A total of 60 Implants (B&B Dental, San Benedetto, BO, Italy) Ø 4.0 × 10 mm-long implants were placed in 10 fresh pig ribs (Figure 1).

All the surgical procedures were performed by one expert surgeon (F.A.).

### 2.1. Selection of Specimens

**Figure 2:**
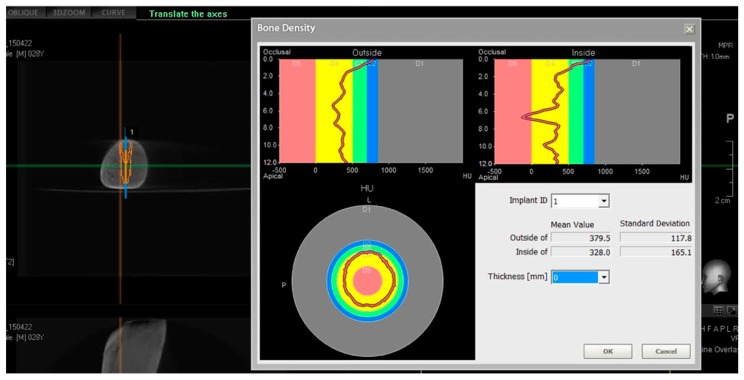
Scanning of the ribs with CBCT scan (Pax i-3d Green, Vatech, Yongin, Korea) to evaluate bone density and select implant site, using Hounsfield measurement unit (Density of 750 < HU).

Fresh pig ribs, not frozen but immediately used for the study, were selected from a slaughterhouse. The soft tissues were carefully dissected and the bone was exposed. Each rib was scanned with CBCT scan (Pax i-3d Green, Vatech, Yongin, Korea) to evaluate bone density, using the Hounsfield measurement unit (HU) (Figure 2). From a sample of 30 pig ribs, we selected 10 that met the inclusion criteria of our study.

### 2.2. Each Selected Site Had to Show

(1) Density of 750 < HU [12], to simulate type III and IV human bone (according to the Lekholm–Zarb classification) [13].
(2) Bone height ≥12 mm.
(3) Bone thickness ≥6 mm.

### 2.3. Surgical Procedures

Each pig rib was fixed on a bench vice in order to avoid micro-movements during surgical procedures and measure recording. The placement and the preparation of each implant site on the pig ribs was randomly extracted using closed envelopes.

### 2.4. Twist Drills Protocol

**Figure 3:**
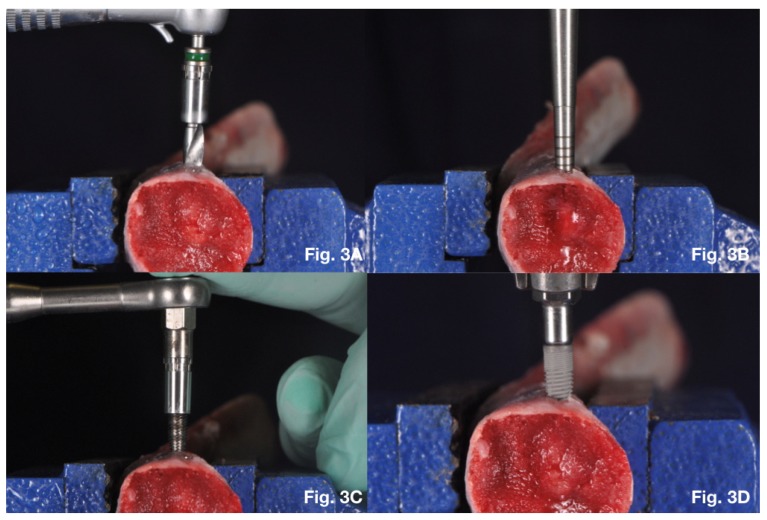
(**A**) Twist drills implant site preparation; (**B**) Summers osteotomes implant site preparation; (**C**) Bone compactors implant site preparation; (**D**) Implant placement using MGT-12 digital torque gauge.

Twenty 4.0 × 10 mm implants were inserted into each pig rib. In this group the implant site was prepared through commercially available surgical burs (B&B Dental, San Benedetto, BO, Italy) (Figure 3A). The standard placement protocol was performed as recommended by the manufacturer. Drilling procedure was started using an in-and-out movement without stopping the hand-piece motor until the drill reached the desired depth level. The first drill was the pilot drill, then 2.2 mm diameter drill, 3.0 mm diameter drill, 3.5 mm diameter drill and at the end with the 4.0 mm diameter drill.

### 2.5. Summers Osteotomes Protocol

Twenty 4.0 × 10 mm implants were inserted into each pig rib. The implant site was prepared by a pilot drill (1.7 mm) followed by Summers osteotomes sizes I, II, III and IV (respectively 2 mm diameter for the osteotome I, 2.5 mm diameter for the osteotome II, 3 mm diameter for the osteotome III and 3.5 mm diameter for the osteotome IV) (Figure 3B).

### 2.6. Bone Compactors Protocol

Twenty 4.0 × 10 mm implants were inserted into each pig rib. In this group the implants sites were prepared as recommended by the manufacturer using bone compactors with increasing diameter. The implant site was performed with a rotationally movement until the device reached the desired depth level. Osteotomy size for 3P Implant B&B Dental (4.0 Ø × 10 mm length) started with the 1.7 mm diameter pilot drill, followed by the 2.2 mm diameter tapered bone compactor, then the 3.0 mm diameter tapered bone compactor, then the 3.5 mm diameter tapered bone compactor and finished with the 4.0 mm diameter tapered bone compactor (Figure 3C).

### 2.7. Analysis of Primary Implant Stability

Implants were inserted with their coronal portion 1 mm inside the surrounding bone according to the manufacturers’ recommendations at the end of the surgical site preparation. The implants were manually placed using the MGT-12 digital torque gauge (Mark-10 Corp, New York, NY, USA) instrument increasing gradually the clockwise force (0.5 mm min^−1^) (Figure 3D).

The peak of insertion torque (PIT) was recorded in Ncm during this procedure.

**Figure 4:**
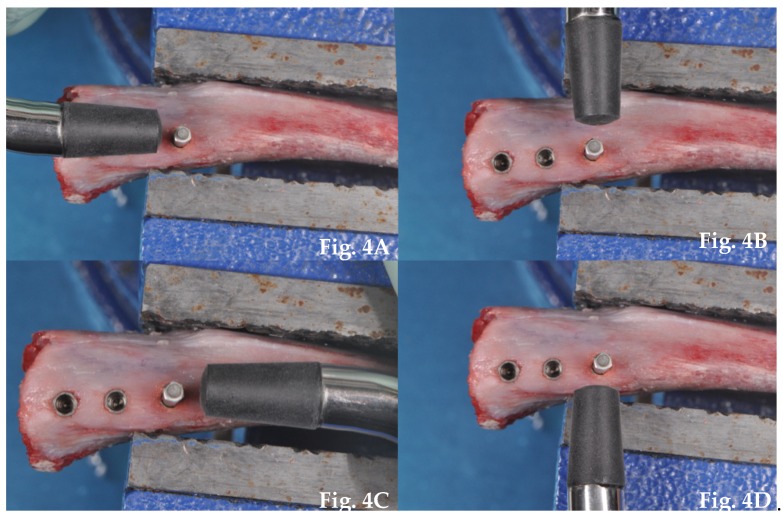
Resonance frequency analysis (RFA) performed with an OSSTELL ISQ™ device; (**A**) Measurement of ISQ D-V; (**B**) Measurement of ISQ M-V; (**C**) Measurement of ISQ M-L; (**D**) Measurement of ISQ D-L.

ISQ was determined by resonance frequency analysis with the OSSTELL ISQ™ device (Integration Diagnostics AB, Göteborg, Sweden). The resonance frequency was measured using a customized SmartPeg with a no contacting technique. The SmartPeg was screwed inside the implant and magnetic waves produced by OSTELL ISQ™ simulated the stress of the prosthetic load. The probe was laterally oriented in relation to the transducer and four measurements of ISQ (M-V, D-V, M-L, D-L) were recorded for each fixture (Figure 4). Each measurement was repeated at least twice and the highest value was taken as reference for the statistical analysis.

All implants were unscrewed to measure the peak removal torque (PRT). It was measured using the MGT-12 digital torque gauge applying a counter-clockwise gradual torque force (0.5 mm min^−1^) till the displacement of the implant.

**Figure 5:**
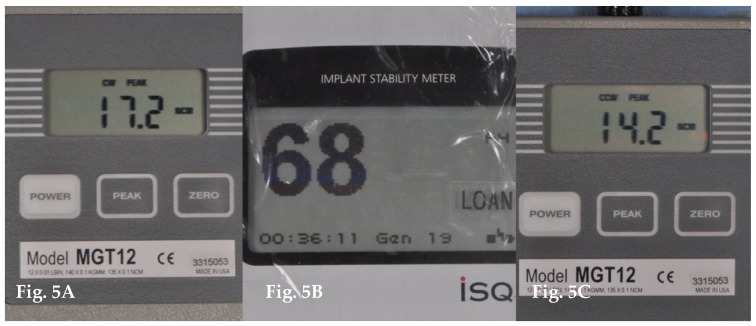
(**A**) Data recording of Peak insertion torque (PIT); (**B**) Data recording of Resonance frequency analysis (ISQ); (**C**) Data recording of Peak removal torque (PRT).

All data were recorded by an independent operator (A.A) (Figure 5).

#### Statistical Analysis

Statistical analysis was performed using the software SPSS Statistics (IBM, SPSS Statistic, Westlands Road, Quarray Bay, Hong Kong, China). Multiple comparisons and differences among groups were evaluated by using an analysis of variance (ANOVA) and a Tukey post hoc t-test, as appropriate. In particular differences between the techniques were compared by one-way ANOVA and, when analysis was significant, the Tukey post hoc t-test was applied. Significant differences were assumed to be at *p* < 0.05.

We computed the minimum sample size with respect to one-way ANOVA, considering: a difference between the three groups; |f| >40%; type 1 error probability alpha = 0.05 and statistical power defined as 1-type 2 error probability beta = 80%.

## 3. Results

A total of 60 implants were placed in 10 pig’s ribs using three different methods of site preparation: 20 implant sites were prepared with twist drills, 20 implant sites with Summers Osteotomes and 20 implant sites with B&B bone compactors. For each implant was measured the value of insertion torque, ISQ and removal torque (Table 1).

For the group of implants placed using twist drills protocol the mean insertion torque was 32.14 ± 8.27 Ncm, the mean ISQ was 69.95 ± 3.57, the mean removal torque was 29.84 ± 9.48 Ncm (Table 2).

In the group of implants inserted performing Summers osteotomes technique the mean insertion torque was 36.94 ± 9.3 Ncm, the mean ISQ was 71.25 ± 4.66, the mean removal torque was 31.92 ± 11.42 Ncm (Table 2).

Implants placed using B&B bone compactor method showed mean insertion torque 40.855 ± 9.57 Ncm, mean ISQ 74.9 ± 3.51 and mean removal torque 37.83 ± 8.98 Ncm (Table 2).

The one-way ANOVA revealed statistically significant differences in insertion torque between three different groups (*p* = 0.013) (Table 2). The one-way ANOVA showed statistically significant differences in ISQ between the three different groups (*p* = 0.0001) (Table 2). The one-way ANOVA evidenced also statistically significant differences in removal torque between three different groups (*p* = 0.039) (Table 2).

**Figure 6:**
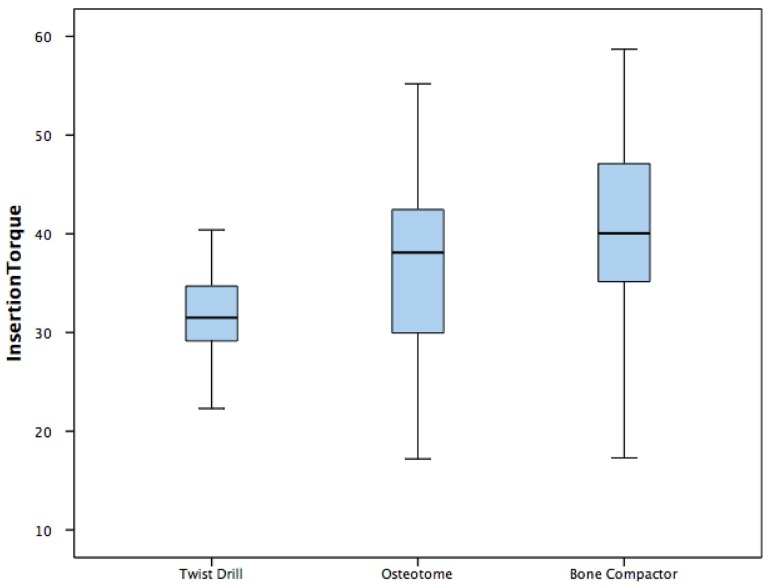
Box plot—Insertion torque.

**Figure 7:**
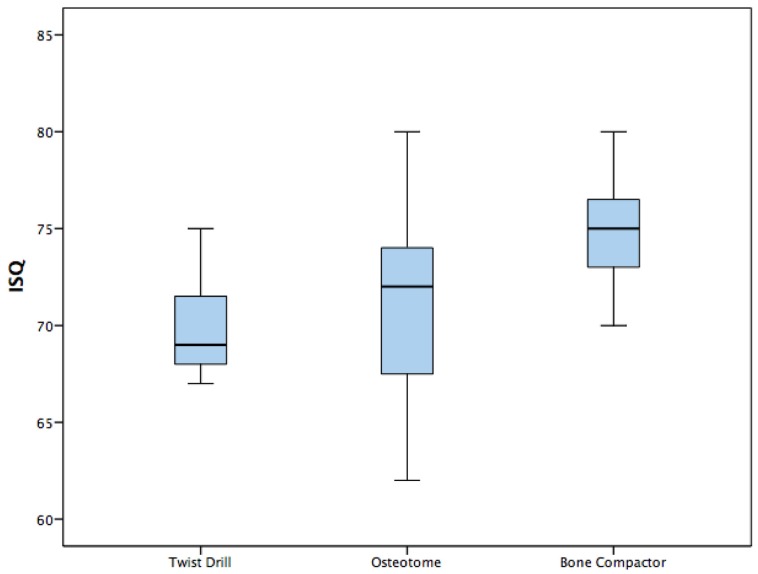
Box plot—Implant stability quotient (ISQ).

**Figure 8:**
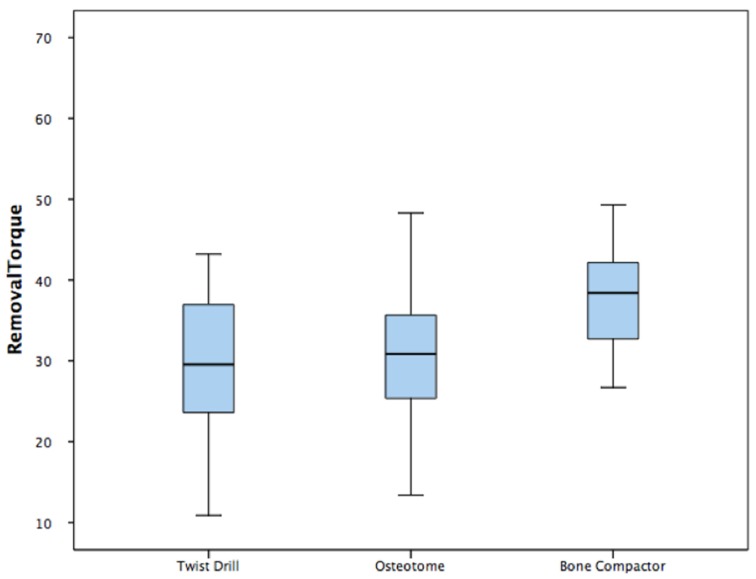
Box plot—Removal torque.

Differences are showed in the Box plots (Figure 6, Figure 7 and Figure 8).

A post hoc Tukey showed a statistically significant difference between the twist drill and bone compactor for insertion torque, a statistically significant difference between the twist drill and bone compactor for ISQ, a statistically significant difference between the osteotome and bone compactor for ISQ, and a statistically significant difference between the twist drill and bone compactor for removal torque (Table 3).

## 4. Discussion

Bone availability is one of the most important parameters in dental implantology [14]. In the past, bone availability was not managed in any way and the prosthetic implant plan had to adapt to the basal conditions of the residual bone. With the improvement of surgical techniques, materials and knowledge, the implant plan evolved from an anatomically guided procedure to a prosthetically guided procedure [15]. Over the years, surgeons became able to place fixtures in zones with enough bone to ensure the long-term success of the implant treatment. The bone height and density changes according to the patient’s age and the edentulous area to be rehabilitated. Usually the area of the jaws with greater bone density is the anterior area of the lower jaw, followed by the front of the upper jaw, then the posterior area of the lower jaw and the posterior area of the upper jaw [3]. In a study by Herrmann et al. the highest implant failure rates were found in the posterior area of the lower jaw, where the bone is usually medullary [16]. However, many studies in the literature have demonstrated how surgery and bone density play a main role the in the rehabilitation of edentulous zones [17]. Engquist et al., in a retrospective multi-centric study, and Friberg et al., in a study of more than 400 implants, observed that the majority of implant failures occurred in low-quality bone or in reabsorbed upper jaws with soft bone (respectively 78% and 66%) [18,19].

The objective of this study was to evaluate any advantages of the osteotomy preparation technique performed with B&B bone compactors compared to with two traditional osteotomy preparation techniques—Twist drills and Summers osteotomes—analysing primary stability parameters in trabecular bone. The insertion torque values were evaluated, the resonance frequency analysis (RFA) and the removal torque values; these variables today, represent the gold standard for primary stability assessment [20]. These parameters, as well as bone density, surgical technique and implant design, represent the best predictive values to obtain implant treatment success [21].

In our study, the choice of fresh pig ribs was made on the literature experiences [22,23]. Pig ribs were selected due to their lower dense cortical portion compared to other animal samples. These ribs simulated the model of a toothless human jaw, thanks to their heterogeneous composition. As evidenced in other experimental studies, the most distal region of the rib is smaller in diameter and has a great percentage of bone marrow. The CT scans confirmed that this animal model has a bone density similar to human type III and IV [12].

In order to avoid early implant failure caused by excessive stress to the bone-implant interface, it is necessary to evaluate a correct planning of the treatment. Several authors showed that an accurate presurgical evaluation of bone density could lead to a better predictability of the bone-implant contact (BIC). Capparè et al. demonstrated the presence of a statistically significant correlation among BIC and insertion torque, showing that bone density has a main role in the initial bone-implant contact [6]. Misch has noted a correlation between BIC and bone density, not only in the first surgical phase but also in following phases, up to the initial prostheses load [24]. In particular, slightly dense bone (D4) offers a smaller BIC percentage in comparison to a D1.

Early implant failure can rise critically if the primary implant stability is inadequate [25]. In the presence of poor bone quality or low primary implant stability, immediate loading protocols are not recommended. To enhance primary implant stability in low-density bone, several surgical protocols have been proposed [26]. Some authors suggested under-sizing the osteotomy implant site with respect to the implant diameter by approximately 10%, in order to reduce bone cutting and improve primary implant stability [27]. Although this procedure increased implant stability, it was not able to modify the bone volume percentage around the implant as compared with non-undersized sites [28]. Another surgical procedure performed to modify bone density surrounding dental implant employed the use of Summers osteotomes. As showed by Glauser et al., this procedure improves the success rate of implants in type IV bone [29].

The BC technique is an osteotome surgical technique used for the dilation and condensation of the bone during implant site preparation. During this procedure, it is possible to obtain bone expansion avoiding dehiscence or fracture of the bone cortex. It is a good alternative to the Summers osteotomes due the ability to perform a lateral bone condensation, differently to the apical expansion of the edentulous ridge in the Summers technique [30,31]. The bone compactors are driven into the bone manually with a straight surgical driver or with a torque ratchet expanding gradually the cancellous bone and improving bone density. This highly predictable procedure avoids the typical trauma of the SO and the discomfort for the patient.

In recent years, several authors investigated the possible interactions between implant site preparation and temperature developed during surgery. Di Fiore et al. evaluated continuous and intermittent techniques showing no differences in terms of overheating [32]; Scarano et al. investigated different shapes of drills. Their study showed that drill geometry seems to be an important factor in heat generation during implant site preparation [33]. In our study, we did not analyse temperature during the surgery, because the osteotomy techniques were not comparable with each other. In fact, while the drill technique is a mechanical procedure, the Summers technique and Bone compaction are both manual. Moreover, overheating was not considered as one of our studied parameters.

The PIT of the implant is one of the main parameters [34,35] to successfully evaluate the implant stability. Analysing our data, implants inserted using this new BC method showed statistically higher biomechanical values than implants placed by Twist drills. This bone condensation technique allows us to enhance the primary implant stability, as demonstrated by the high PIT value in the test group in this study. There is no significant statistical difference for PIT in the SO technique compared to the BC technique. Other parameters that are directly correlated to the implant stability are ISQ and PRT. In this study, the greater ISQ values in the BC technique compared to the other procedures showed a significant increase in the stiffness at the implant–bone interface, especially in the crestal region [20,36,37]. The BC compactor technique showed greater values of PRT than the twist drill technique, expressing a significant amount of bone compaction during the surgical procedures. This bone compaction protocol constitutes a less invasive surgery, with less heat generation and low morbidity and costs. The management of the posterior zones of the jaws, for an implant rehabilitation, could have predictable results and better cost/benefit ratio with BC technique.

## 5. Conclusions

Within the limitations of this study, our in vitro results demonstrate that the bone compactor surgical technique is a good alternative to other surgical protocols for implant placement in medullary bone. This procedure allows us to improve primary implant stability through lateral bone compression, and to perform gradual and controlled forces during implant site preparation. The use of thread formers of increasing diameters with torque rachet allows for non-invasive and controlled bone expansion. In vivo studies and long-term data with success rates will be necessary to evaluate the predictability of this procedure.

## Figures and Tables

**Table 1:** Primary stability data of each group.

	Insertion Torque	RFA	Removal Torque		Insertion Torque	RFA	Removal Torque		Insertion Torque	RFA	Removal Torque
1	22.4	68	23.4	1	24.8	72	23.8	1	33.2	73	38.4
2	46.3	74	42.2	2	47.1	78	66.8	2	53.1	75	44.9
3	22.3	67	10.9	3	25	65	26.9	3	29	75	30.3
4	30.5	71	21.6	4	29.5	65	13.4	4	37.5	65	26.7
5	44.5	68	29.9	5	44	62	33.9	5	46.9	70	40.7
6	40.4	79	37.7	6	39.9	65	30.2	6	47.2	78	47.3
7	32.7	68	43.2	7	38.2	72	23.8	7	37.2	73	38.4
8	34.3	72	36.2	8	45.9	75	35.9	8	47	75	49.3
9	32.1	69	30.9	9	47.9	80	32.1	9	55.4	77	39.5
10	29.9	67	22.8	10	35.6	74	29.2	10	39.9	75	36.2
11	29.2	68	27.8	11	28.3	67	30.3	11	36.2	76	31.3
12	35.1	69	28.8	12	40.9	72	35.9	12	41.7	82	43.6
13	29.7	69	29.2	13	38	73	31.4	13	48.9	76	37.2
14	48.2	70	43.1	14	55.2	74	48.3	14	58.7	77	57
15	32	67	31	15	38.3	71	35.4	15	40.2	73	38.2
16	29.1	62	27.8	16	35.7	72	34.6	16	34.1	75	34.1
17	31	70	43.2	17	36.2	73	23.8	17	39.9	75	38.4
18	34.2	72	32.2	18	40.1	76	38.6	18	40.5	80	40
19	14.1	75	11.2	19	17.2	68	14.2	19	17.3	75	14.2
20	24.8	68	23.8	20	30.4	71	30	20	33.2	73	31

**Table 2:** Mean and Standard Deviation of Insertion Torque, ISQ and Removal Torque of each group and results of Two-way ANOVA test.

Variable	Twist Drills (TD)	Summers Osteotomes (SO)	Bone Compactors (BC)	P (Anova)
**Insertion Torque (Ncm)**	32.14 ± 8.27	36.94 ± 9.3	40.85 ± 9.57	0.013 *
**RFA (ISQ)**	69.95 ± 3.57	71.25 ± 4.66	74.9 ± 3.51	0.0001 *
**Removal Torque (Ncm)**	29.84 ± 9.48	31.92 ± 11.42	37.83 ± 8.98	0.039 *

*p* < 0.05; * Statistically significant differences *p* < 0.05.

**Table 3:** Results of Post hoc Tukey test.

Variable		P (Post Hoc Tukey)
**Insertion Torque**	Twist Drill vs Osteotome Twist Drill vs Bone Compactor Osteotome vs Bone Compactor	0.226 0.01 * 0.36
**ISQ**	Twist Drill vs Osteotome Twist Drill vs Bone Compactor Osteotome vs Bone Compactor	0.41 0.0001 * 0.014 *
**Removal Torque**	Twist Drill vs Osteotome Twist Drill vs Bone Compactor Osteotome vs Bone Compactor	0.79 0.038 * 0.16

* Statistically significant differences *p* < 0.05.

## Abbreviations

PS	primary implant stability
PIT	peak insertion torque
RFA	resonance frequency analysis
ISQ	implant stability quotient
PRT	peak removal torque
